# *SQSTM1/p62* Knockout by Using the CRISPR/Cas9 System Inhibits Migration and Invasion of Hepatocellular Carcinoma

**DOI:** 10.3390/cells12091238

**Published:** 2023-04-25

**Authors:** Jinghua Lu, Yipei Ding, Wanqiu Zhang, Yuanyuan Qi, Jin Zhou, Naihan Xu, Yaou Zhang, Weidong Xie

**Affiliations:** 1 State Key Laboratory of Chemical Oncogenomics, Shenzhen International Graduate School, Tsinghua University, Shenzhen 518055, China; 2 Shenzhen Key Laboratory of Health Science and Technology, Shenzhen International Graduate School, Tsinghua University, Shenzhen 518055, China; 3 Institute for Ocean Engineering, Shenzhen International Graduate School, Tsinghua University, Shenzhen 518055, China

**Keywords:** migration, invasion, hepatocellular carcinoma, SQSTM1/p62

## Abstract

Migration and invasion play crucial roles in the progression of hepatocellular carcinoma (HCC), but the underlying mechanisms are not clear. Analysis of clinical samples indicates that SQSTM1/p62 is highly expressed in HCC and seriously affects the prognosis of patients. Subsequently, we showed that *SQSTM1/p62* knockout using the CRISPR/Cas9 system led to impaired migration and invasion of HCC, upregulated Keap1, and promoted the inhibitory effect of Keap1 on Nrf2. Then, the inactivation of Nrf2 inhibited the expression of matrix metalloproteinases (MMPs), thus attenuating the migration and invasion of HCC. We also found that *SQSTM1/p62* knockout significantly inhibited migration and invasion in a lung metastasis model of nude mice with HCC. Furthermore, we found that cisplatin not only significantly inhibited the expression of SQSTM1/p62 but also slowed down the migration and invasion of HCC, while the inflammatory microenvironment accelerated the migration and invasion of HCC. These results suggest for the first time that *SQSTM1/p62* knockout inhibits the migration and invasion of HCC through the Keap1/Nrf2/MMP2 signaling pathway. SQSTM1/p62 may be developed into a key drug target to regulate the migration and invasion of HCC cells.

## 1. Introduction

HCC is one of the deadliest tumors [1]. China has a high incidence of HCC, and the overall survival rate is unsatisfactory [2,3]. Many clinical studies have shown that migration and invasion are the main reasons for this [4,5]. Migration and invasion are two major characteristics of HCC progression and recurrence. The cellular biological process is generally as follows: the primary tumor locally invades the extracellular matrix (ECM), then penetrates the basement membrane and the vascular endothelium, enters the blood, and, through circulation, reaches distant organs where it forms metastatic tumors [6,7]. In general, migration and invasion largely determine the success or failure of HCC treatments [8]. Therefore, it is crucial to explore the mechanisms of the migration and invasion of HCC cells and identify effective targets.

The human *p62* gene is located on chromosome 5 [9] and encodes a p62 protein with 440 amino acid residues, also known as *SQSTM1* [10]. SQSTM1/p62 has multiple functional domains and is closely related to protein ubiquitination, selective autophagy, oxidative stress, and other reactions [11,12]. In recent years, several studies have confirmed that SQSTM1/p62 is overexpressed in various cancers [13]. Increasing evidence shows that SQSTM1/p62 can affect the migration and invasion of tumors in a variety of ways [13,14,15,16]. However, the molecular mechanisms of SQSTM1/p62 involvement in migration and invasion are still unclear. Previous studies have shown that SQSTM1/p62 can release Nrf2 by combining with Keap1 [17,18]. In HCC, abnormal activation of Nrf2 promotes the expression of MMP2 and MMP9 [19], which are matrix metalloproteinases that can degrade the basement membrane and promote tumor migration and invasion [20,21].

Cancer is also closely associated with inflammation. The inflammatory microenvironment favors tumor progression by accelerating tumor migration and invasion [22,23,24,25]. Several studies have reported that SQSTM1/p62 is significantly upregulated in the inflammatory microenvironment [26,27,28,29]. Therefore, it is important to further explore the effects of SQSTM1/p62 on the migration and invasion of HCC cells in the inflammatory microenvironment. Cisplatin, also known as diamminedichloroplatinum (DDP), is an effective drug for controlling tumor cell proliferation and inhibiting tumor cell migration and invasion [30,31]. In addition, DDP treatment significantly reduces the levels of SQSTM1/p62. Taken together, these results indicate that SQSTM1/p62 is regulated by many factors. However, it is not clear whether these environmental or pharmacological regulations of SQSTM1/p62 levels affect the migration and invasion of HCC.

The CRISPR/Cas9 system is a new and rapidly developing gene-editing technology. Gene editing systems are typically used to examine gene function and identify drug targets. In previous studies, *SQSTM1/p62* knockout was performed using CRISPR/Cas9 [32,33]. However, most of these studies have focused on autophagy. There is no record of *SQSTM1/p62* knockout and functional investigation into migration and invasion using the CRISPR/Cas9 system. In this study, we generated *SQSTM1/p62* wild-type HepG2 cells (*SQSTM1* WT HepG2 cells) and *SQSTM1/p62* knockout HepG2 cells (*SQSTM1* KO HepG2 cells) using the CRISPR/Cas9 system. The newly developed *SQSTM1/p62* knockout HepG2 cells may provide an important source for future research. We then studied the effects of *SQSTM1/p62* knockout on the migration and invasion of HCC cells in vivo and in vitro and investigated how SQSTM1/p62 regulates the migration and invasion of HCC through the Keap1/Nrf2/MMP2 signaling pathway. We also investigated the influence of the induced inflammatory microenvironment and antitumor drug DDP on the migration and invasion of HCC in the presence of SQSTM1/p62.

## 2. Materials and Methods

### 2.1. Bioinformatics Analysis

The Cancer Genome Atlas (TCGA) is a database that can provide important information about the mechanisms of cancer occurrence and development. TIMER2.0 (http://timer.cistrome.org/, accessed on 25 July 2021) provides visualization functions [34]. Using TIMER2.0, we mined the data of the expression of *SQSTM1/p62* in all forms of cancer in the TCGA database and evaluated the potential of this gene as a therapeutic target (*p* < 0.001). The online analysis database Ualcan (http://ualcan.path.uab.edu/index.html, accessed on 5 September 2021) and the powerful tool GEPIA2 (http://gepia2.cancer-pku.cn/#index, accessed on 18 September 2021) [35] were used to analyze the SQSTM1/p62 expression profile data in human liver cancer tissues and normal human liver tissues in TCGA and GTEx databases. Finally, SQSTM1/p62 immunohistochemical data and prognosis analyses were obtained from The Human Protein Atlas (HPA) (https://www.proteinatlas.org, accessed on 27 September 2021).

### 2.2. Cell Culture and Treatment

HEK293T cells were supplied by the American Type Culture Collection (Manassas, VA, USA), HepG2 cells were provided by the Cell Bank of the Chinese Academy of Sciences (Shanghai, China), and THP-1 cells were purchased from the Procell Life Science & Technology Co., Ltd. (Wuhan, China). *SQSTM1* WT HepG2 cells, *SQSTM1* KO HepG2 cells, and *SQSTM1* KO2 HepG2 cells were generated using the CRISPR/Cas9 system, as described in the following section. All cells were cultured in Dulbecco’s Modified Eagle Medium (DMEM; Thermo Fisher Scientific, Waltham, MA, USA), which contained 1% penicillin-streptomycin antibiotics (Thermo Fisher Scientific, Waltham, MA, USA), 10% fetal bovine serum (FBS; Pan Biotech, Adenbach, German), and was incubated in a humidified atmosphere of 95% air/5% CO_2_ (vol/vol) at a stable temperature (37 °C) and constant pH (7.2–7.4). According to different experimental requirements, the cells were counted after digestion and inoculated in tissue culture plates (Jet Biofiltration Co., Ltd., Guangzhou, China). DDP (MedChemExpress, MCE, Monmouth Junction, NJ, USA) was added for 24 h after the cells had grown to 60–70% confluence. Lipopolysaccharide (LPS; Sigma-Aldrich, St. Louis, MO, USA) was added for 0–24 h after the cells had grown to 50–60% confluence to simulate the inflammatory microenvironment.

### 2.3. Generation of SQSTM1/p62 KO HepG2 Cells by CRISPR/Cas9 System

*SQSTM1/p62* KO in HepG2 Cells was generated using the CRISPR/Cas9 system, as previously described, with slight modifications [36]. First, the plasmid pLX-sgRNA (Addgene, Watertown, MA, USA) was expressed in *Escherichia coli* (*E. coli*) Stbl3 glycerol strain (Beyotime, Shanghai, China) and extracted using the TIANprep mini plasmid kit (Tiangen, Beijing, China) for amplification. Then, it was immediately used as the template to conduct multiple extension PCR with sgRNA-p62-2, sgRNA-p62-3, sgRNA-p62-8, and sgRNA-p62-10 (Table A1), and the target PCR products were obtained. Subsequently, pLX-sgRNA and target PCR products were digested with the restriction endonuclease *Xho* I and *Nhe* l (TaKaRa, Mountain View, CA, USA) and retrieved using an agarose recovery kit (Tiangen, Beijing, China) after 0.8–1% agarose gel electrophoresis. Then, pLX-sgRNA was linked to the target fragments (460 bp) using T4 ligase, and recombinant plasmids were obtained.

Second, the recombinant plasmids (plx-sg2, plx-sg3, plx-sg8, plx-sg10) and pLX-sgRNA were transformed into *E. coli* DH5α (TaKaRa, Mountain View, CA, USA) and screened using 50 μg/mL ampicillin (AMP). Single colonies were selected from the plx-sg2, plx-sg3, plx-sg8, and plx-sg10 groups for amplification and gene sequencing. Single positive colonies with the correct sequencing results were selected for plasmid amplification.

Third, the encapsulated pCV-VSVg, packaged pMDLg/pRRE, and pRSV-Rev and pCW-Cas9 (Addgene, Watertown, MA, USA) were mixed and transfected into 293T cells to complete virus packaging, and the virus supernatant was collected. When HepG2 cells had grown to 50–70% confluence, a mixture of virus supernatant from 293T cells and DMEM (1:1) was added to HepG2 cells, which were infected with the virus for 3 h, and then screened continuously using 1–2 μg/mL puromycin (MCE, USA). Finally, HepG2-cas9 cells that produce Cas9 were generated.

Lastly, we delivered pLX-sgRNA empty and recombinant plasmids to HepG2-cas9 cells and constructed a HepG2-pLX-Cas9 cell line. Briefly, the encapsulated pCW-VSVg, packaged pMDLg/pRRE, and pRSV-Rev were mixed with empty pLX-sgRNA and recombinant plasmids (plx-sg2, plx-sg3, plx-sg8, plx-sg10) and transfected into 293T cells. After virus packaging, HepG2-Cas9 cells were infected with the virus solution as described above and screened using blasticidin (Genomeditech, Shanghai, China). Finally, the HepG2-pLX-Cas9 cell line, which expressed both sgRNA and Cas9, was obtained. Cas9 was induced using 2 μg/mL doxycycline (MCE, USA) for *SQSTM1/p62* gene editing. The expression of SQSTM1/p62 in different cell types was determined using Western blotting (WB). Soon afterward, the cells with a significant decrease in SQSTM1/p62 expression levels were selected and underwent multiple cycles of enrichment using the single-cell culture method, and clones of *SQSTM1/p62* KO HepG2 cells were obtained.

### 2.4. MTT Assay

A thiazolyl blue tetrazolium bromide (MTT; Sangon Biotech, Shanghai, China) assay was used to determine cell growth. Briefly, *SQSTM1* WT HepG2 cells and *SQSTM1* KO HepG2 cells were inoculated into 96-well plates in equal numbers with DMEM containing 1% FBS, and five culture plates were inoculated in total. After 6 h, cells were incubated with DMEM containing 10% FBS for 0, 24, 48, 72, or 96 h. Subsequently, 20 μL sterilized MTT (5 mg/mL) were added to each well and incubated for 3 h at 37 °C. Finally, the medium was carefully removed, 100 µL dimethyl sulfoxide solution were added to fully dissolve the formazan, and OD_490nm_ was measured using a spectrophotometer. The cytotoxicity of LPS or DDP was also analyzed using the MTT assay. Briefly, *SQSTM1* WT HepG2 cells and *SQSTM1* KO HepG2 cells were inoculated into 96-well plates in equal numbers with DMEM containing 10% FBS and LPS (1 μg/mL) or DDP (5 µM or 10 µM). After 24 h, cell growth was determined as described above.

### 2.5. Wound Healing Assay

The wound healing assay, also known as the scratch experiment, is a basic way to study cell migration and directly observe this dynamic process. Briefly, when the cells grew to a density of more than 90%, straight and regular wounds were made in the cells using a sterilized toothpick. The cells were rinsed with phosphate-buffered solution (PBS) and then cultured in DMEM containing 1% FBS for 48 h. Images at 50×/100× magnification were captured under a microscope at 0, 24, and 48 h, and the scratch width and cell migration rate were calculated.

### 2.6. Transwell Migration Assay

When *SQSTM1* WT HepG2 cells and *SQSTM1* KO HepG2 cells were in the logarithmic growth period, they were inoculated into Transwell inserts with a pore size of 8 μm (Jet Biofiltration Co., Ltd., Guangzhou, China) at the same density with 200 μL serum-free medium (SFM). The Transwell inserts were then placed in 24-well plates filled with 600 μL DMEM and 20% FBS and cultured in a humidified tissue culture incubator at 37 °C in a 5% CO_2_ atmosphere for 24 h. The cells were then fixed in 4% paraformaldehyde for 20 min in the dark and stained with 0.1% crystal violet for 30 min in the dark. Finally, the unmigrated cells were washed under slow water flow and wiped off with a cotton swab. After air-drying, images were captured at 200× magnification under a microscope. The number of migrating cells was measured using ImageJ software (National Institutes of Health, Bethesda, MD, USA).

### 2.7. Transwell Invasion Assay

The invasive ability and metastatic potential of *SQSTM1* WT HepG2 cells and *SQSTM1* KO HepG2 cells were assessed using Corning^®^ BioCoat™ Matrigel^®^ Invasion Chambers with a pore size of 8 μm (Corning^®^ BioCoat™, Bedford, MA, USA). Briefly, warm SFM (37 °C) was added to the interior of the 24-well Matrigel^®^ Invasion Chambers and the bottom of the wells for 2 h to rehydrate the matrigel. After rehydration, the 24-well Matrigel^®^ Invasion Chambers were transferred to the wells containing DMEM with 20% FBS, *SQSTM1* WT HepG2 cells, and *SQSTM1* KO HepG2 cells and were inoculated into the chambers at the same density in SFM. Immediately, the Matrigel Invasion Chambers were incubated for 24 h in a humidified atmosphere of 5% CO_2_ at 37 °C, followed by the Transwell migration assay.

### 2.8. Quantitative Real-Time Polymerase Chain Reaction (RT-PCR) Assay

Total cellular RNA was extracted using RNAiso Plus (TaKaRa Biotechnology, Dalian, China), according to previous studies [37,38]. Total RNA (500 ng) was immediately used to synthesize cDNA using the *Evo M-MLV* RT Mix Kit (Accurate Biology, Changsha, China) according to the manufacturer’s instructions, and then the expression levels of mRNAs were quantitatively analyzed with the SYBR^®^ Green Premix Pro Taq HS qPCR Kit (Accurate Biology, Changsha, China). Finally, relative gene expression was normalized to β-actin and calculated using the 2^−ΔΔCt^ method. The target primers were synthesized by GENEWIZ (Cambridge, MA, USA) and are as follows: h-β-actin-F/R: CATGTACGTTGCTATCCAGGC/CTCCTTAATGTCACGCACGAT; h-NFE2L2-F/R: TCCAGTCAGAAACCAGTGGAT/GAATGTCTGCGCCAAAAGC-TG; h-MMP2-F/R: CCCATGCGGTTTTCTCGAAT/CAAAGGGGTATCCATCGCCAT; h-MMP9-F/R: AGACCTGGGCAGATTCCAAAC/CGGCAAGTCTTCCGAGTAGT; h-CD11b-F/R: CAGCCTTTGACCTTATGTCATGG/CCTGTGCTGTAGTCGCACT; h-CD68-F/R: CGAGCATCATTCTTTCACCAGCT/ATGAGAGGCAGCAAGATGGACC.

### 2.9. Western Blotting Assay

Western blotting analysis was performed as previously described with slight modifications [37,38]. Briefly, the treated cells were lysed with lysis buffer, collected, and centrifuged, and the total protein concentration was measured using the Coomassie Brilliant Blue G250 (Beyotime, Shanghai, China) method. Subsequently, heat-denatured protein samples were separated using 10% (g/mL) sodium dodecyl sulfate-polyacrylamide gel electrophoresis (SDS-PAGE) at 135 V for 2 h and then transferred (200 mA, 2 h) to nitrocellulose membranes (NC; Pall, NY, USA) in an ice bath. Immediately, the membranes were blocked using 5% (g/mL) defeat dried milk for 2 h at 25 °C and then probed with primary antibodies overnight at 4 °C. After washing four times (10 min/wash) with TBST buffer (TBS with 0.5% Tween-20), the membranes were incubated with the respective secondary antibody for 1.5 h at 25 °C. After washing three times (15 min each time) with TBST, the protein blots were visualized using a chemiluminescence solution (Thermo Fisher Scientific, Waltham, MA, USA). Finally, the relative grey density values of the protein blots were quantified using ImageJ software and normalized to the density of β-actin. The following antibodies were used in this study: β-actin (Sigma-Aldrich, A1978, Mouse, 1:5000), SQSTM1/p62 (ABclonal Technology, Wuhan, China, A19700, Rabbit, 1:1000), Keap1 (ABclonal Technology, Wuhan, China, A21724, Rabbit, 1:1000), Nrf2 (ABclonal Technology, Wuhan, China, A0674, Rabbit, 1:1000), MMP2 (GenXspan, AL, USA, GXP328105, Rabbit, 1:1000), MMP9 (Cell Signal Technology, Boston, MA, USA, #13667, Rabbit, 1:1000), mouse secondary antibody (Cell Signal Technology, Boston, MA, USA, #7076S, 1:5000), and rabbit secondary antibody (Cell Signal Technology, Boston, MA, USA, #7074P2, 1:5000).

### 2.10. Gelatin Zymography Assay

Concerning the previous method [39,40], after minor adjustments, the activity of MMP2 was determined by gelatin zymography. Generally, the cells were cultured in 12-well plates in equal numbers. When the cells were grown to a density of 60–70%, they were cultured in 800 μL serum-free DMEM for 24 h. Then, 500 μL of supernatant were collected in ultrafiltration centrifuge tubes (3 kDa, Merck, Darmstadt, Germany), and 30–50 μL concentrated solutions were acquired after centrifugation at 14,000× *g* at 4 °C for 30 min and centrifugation at 2000× *g* and 4 °C for 5 min. Next, 30 μL samples containing 22.5 μL concentrated solutions and 7.5 μL 5× loading buffer (Table A2) were electrophoresed using 10% (g/mL) SDS-PAGE containing 0.1% gelatin (Sangon Biotech, Shanghai, China) at 135 V for 1–2 h. After electrophoresis, the gel was incubated in an eluent (Table A3) and washed twice (40 min/wash) while shaking at a slow speed. Immediately, the gel was washed twice (20 min/time) with a rinsing solution (Table A4) and incubated with fresh incubation buffer (Table A5) at 37 °C for 48 h. Then, Coomassie brilliant blue staining solution (30% Methanol, 10% Acetic Acid, and 0.05% Coomassie Brilliant Blue) was added to the gel at 37 °C for 3 h, and the gel was destained with decolorizing solution (V_Methanol_:V_Acetic acid_:V_Water_ = 5:1:4) while shaking at a slow speed for 1–2 h. Finally, the gel was taken, and the active area of matrix metalloproteinase was displayed as a transparent band under the dark blue background.

### 2.11. siRNA Transfection

Transfection is an effective method for verifying target and signaling pathways. Taking 6-well plates as an example, 5 μL Lipofectamine^®^ 3000 (ThermoFisher Scientific, Waltham, MA, USA) and 245 μL Opti-MEM^®^ (ThermoFisher Scientific, Waltham, MA, USA) were mixed and left to stand for 5 min to obtain the transfection reagent. Then, si-NC (a common negative control without homology to the sequence of the target gene) or siRNA-Target gene (e.g., siRNA-p62 and siRNA-Nrf2; RiboBio, Guangzhou, China) were mixed with 245 μL Opti-MEM^®^, and the siRNA solution was obtained. The two solutions were then mixed and allowed to stand for 20 min. Then, 500 μL Opti-MEM^®^ of the mixed solution were added to each well of cells at 60% confluence, and the final concentration of siRNA remained at 100 nM. After 12–48 h of transfection, the cells were used for Transwell migration, Transwell invasion, gelatin zymography, and RNA and protein assays.

### 2.12. Animal Experiments

BALB/c-nu/nu nude mice [SPF grade, Male, six weeks old, Certified No. SCXK (Guang-dong) 2022-0002], chow diets, and corncob padding were supplied by the Guangdong Medical Laboratory Animal Center (Guangzhou, China). The animals were fed in a closed animal rearing cabinet (F2005001725) in a quarantine room (ADM-024) in a suitable environment (humidity: 40–70%, temperature: 20–26 °C, light illumination, 12 h/day). This study strictly followed the National Institutes of Health Guide for the Care and Use of Laboratory Animals to ensure animal welfare and ethical standards. The protocol was approved by the Bioethics Committee of the Shenzhen International Graduate School of Tsinghua University (Ethics issue (2022) No. F109). In this study, a lung metastasis model of HCC in nude mice was established by tail vein injection. Briefly, nude mice were randomly divided into control and experimental groups based on their weight (*n* = 5 per group). *SQSTM1* WT HepG2 cells (1.5 × 10^6^) and *SQSTM1* KO HepG2 cells (1.5 × 10^6^) were injected into the tail vein of mice in the control and experimental group, respectively. The weight and general condition of the mice were recorded every other day. After 7 weeks of inoculation, all nude mice were killed humanely and dissected to observe metastasis in the lungs and liver. In addition, all lung and liver tissues were weighed, and portions were produced as 4% paraformaldehyde-fixed paraffin-embedded samples for conventional hematoxylin and eosin (H&E) staining and immunohistochemical analysis (MMP2).

### 2.13. Statistical Analysis

In this study, the experimental data were statistically analyzed using GraphPad Prism 8.4.3 (GraphPad Software Inc., Waltham, MA, USA) and are shown as mean ± standard deviation (SD). Differences with statistical significance between different groups were evaluated using one-way ANOVA with Tukey’s post hoc test. Statistical significance was set at *p* < 0.05.

## 3. Results

### 3.1. SQSTM1/p62 Is Overexpressed in HCC Tissues and Seriously Affects the Prognosis

**Figure 1:**
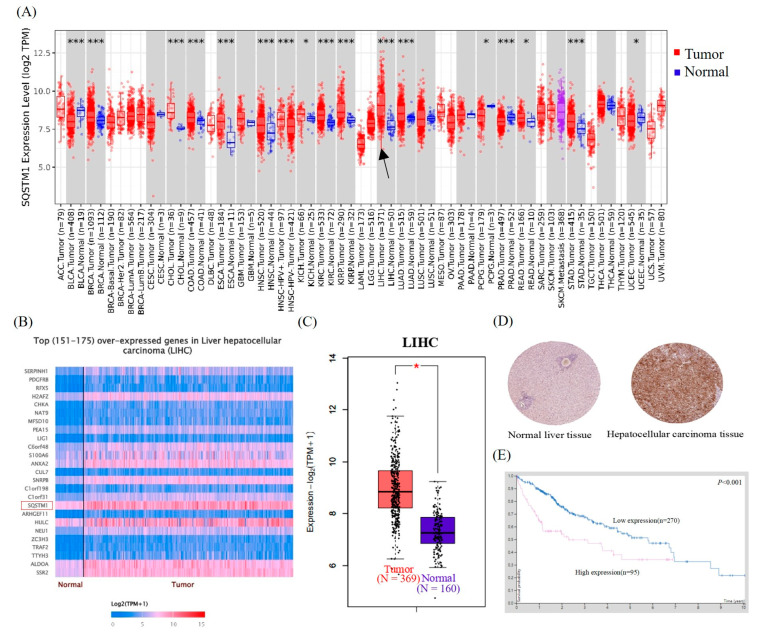
Bioinformatics analysis of SQSTM1/p62 expression and prognosis. (**A**) Expression of the *SQSTM1/p62* gene in various cancers based on the TCGA database. The black arrow emphasizes the expression of SQSTM1/p62 in LIHC tissues and normal liver tissues, * *p* < 0.05, *** *p* < 0.001. (**B**) Analysis of top (151–175) over-expressed genes in HCC based on the TCGA database. (**C**) Differential expression of SQSTM1/p62 mRNA in HCC tissues and normal liver tissues based on TCGA and GTEx databases, red star represents *p* < 0.05. (**D**) Immunohistochemical analysis of SQSTM1/p62 protein in normal liver tissues and HCC tissues based on the HPA database. (**E**) Influence of SQSTM1/p62 on the survival and prognosis of HCC patients based on the HPA database, *p* < 0.001.

In this study, the expression levels of *SQSTM1/p62* in various cancers in the TCGA database were mined using TIMER2.0, and the results showed that *SQSTM1/p62* was usually highly expressed in tumors, especially in HCC tissues (Figure 1A). Therefore, Ualcan was used to analyze the SQSTM1/p62 expression profile data of HCC tissues and normal human liver tissues from TCGA and GTEx databases. As shown in Figure 1B,C, SQSTM1/p62 mRNA levels were significantly upregulated in HCC. In addition, analysis of the HPA database showed that the rate of positive SQSTM1/p62 protein immunohistochemistry staining in HCC tissues was higher than that in normal liver tissues (Figure 1D), and the survival period of patients with high SQSTM1/p62 expression was significantly shorter than for patients with low SQSTM1/p62 expression (Figure 1E). The above clinical data suggest that the expression of SQSTM1/p62 in HCC tissue was significantly higher than that in normal human liver tissue at both the mRNA and protein levels and may contribute to the invasion and metastasis of HCC. Furthermore, some studies have found that the expression level of SQSTM1/p62 in HCC tissue is closely related to tumor size, venous invasion, histological grade, metastasis, and TNM stage (Table A6), further confirming that it may be an effective target for the treatment of HCC migration and invasion [41]. Therefore, we examined the effect of SQSTM1/p62 on the migration and invasion of HCC.

### 3.2. SQSTM1 WT HepG2 Cells and SQSTM1 KO HepG2 Cells Are Generated Using CRISPR/Cas9 System

**Figure 2:**
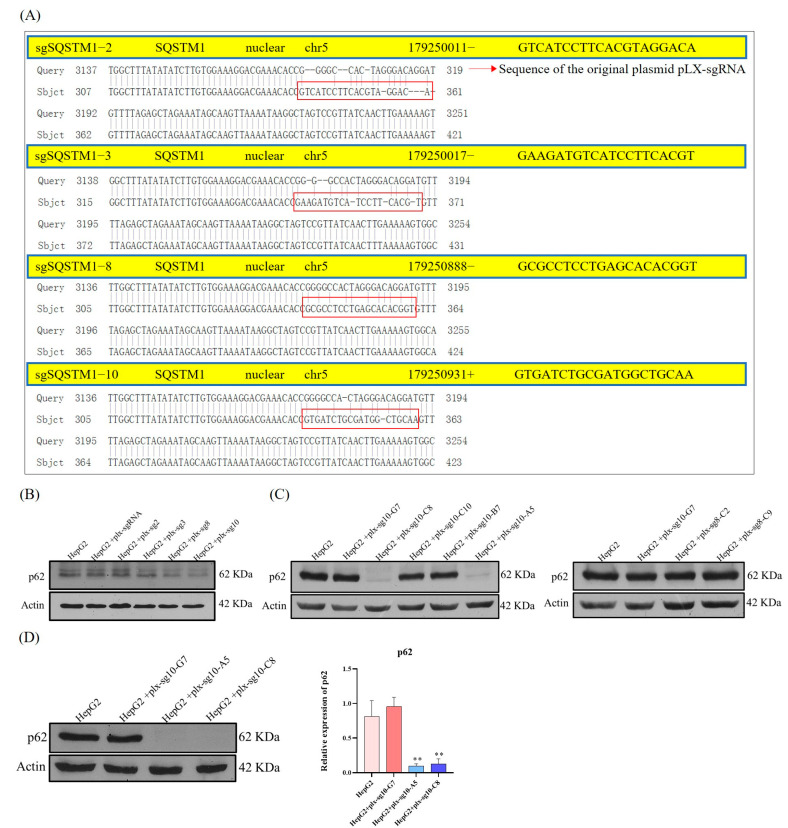
*SQSTM1* WT HepG2 cells and *SQSTM1* KO HepG2 cells were generated using the CRISPR/Cas9 system. (**A**) Gene sequencing results of positive single colonies, in which the sequence in the red box was the correct sequence; that is, there was no gene mutation. (**B**) The expression levels of SQSTM1/p62 in different cells after 7 days of 2 ug/mL doxycycline induction were detected by WB. (**C**) The expression levels of SQSTM1/p62 in purified single cells were detected by WB. Among them, G7\C8\C10\B7\A5\C2\C9 represents the position of sgRNA in the 96-well plate. (**D**) The expression levels of SQSTM1/p62 in HepG2, plx-sg10-G7, plx-sg10-A5, and plx-sg10-C8 cells were verified by WB. All data are shown as mean ± SD (*n* = 3), ** *p* < 0.01.

The CRISPR/Cas9 system is a powerful gene editing technology that has the advantages of the easy design of genomic targets, prediction of missing sites, and the possibility of simultaneously modifying multiple genomic sites [36,42]. To better understand the influence and mechanisms of SQSTM1/p62 on the migration and invasion of human hepatoma cells, *SQSTM1* WT HepG2 cells and *SQSTM1* KO HepG2 cells were successfully generated using the CRISPR/Cas9 system. First, using plasmid pLX-sgRNA as the template, sgRNA-p62-2, sgRNA-p62-3, sgRNA-p62-8, and sgRNA-p62-10 were selected for plasmid recombination, and four recombinant plasmids (plx-sg2, plx-sg3, plx-sg8, plx-sg10) and an empty plasmid pLX-sgRNA were, respectively, transformed into *E. coli* DH5α and screened using Ampicillin. Single colonies of DH5α bacterial strains with Ampicillin resistance were selected. The gene sequencing results (Figure 2A) showed that sgRNA recombination with the empty pLX-sgRNA plasmid was successful. After transfection of these sgRNA plasmids in the Cas9-expressing cell line, the efficiency of *SQSTM1/p62* knockout was assayed using WB. The results (Figure 2B) showed that the expression of SQSTM1/p62 in “HepG2 + plx-sg8” cells and “HepG2 + plx-sg10” cells were significantly decreased. HepG2 + plx-sg8 and “HepG2 + plx-sg10” cells were subjected to several cycles of enrichment using single-cell culture. The results (Figure 2C) showed that *SQSTM1/p62* in “HepG2 + plx-sg10-A5” and “HepG2 + plx-sg10-C8” single cell lines derived from “HepG2 + plx-sg10” cells were almost knocked out but in “HepG2 + plx-sg10-G7” derived from “HepG2 + plx-sg10” cells were not knocked out, suggesting that this cell line was not pure and required further purification. Finally, the purification results (Figure 2D) indicated that the *SQSTM1/p62* in “HepG2 + plx-sg10-A5” and “HepG2 + plx-sg10-C8” single cell lines were completely knocked out. Therefore, after 3–5 rounds of enrichment, these cell lines were selected as *SQSTM1/p62* knockout HepG2 cell lines. For convenience, in the next experiments of this study, the “HepG2 + plx-sg10-C8” and “HepG2 + plx-sg10-A5” single cell lines were selected as the experimental group, named *SQSTM1* KO and *SQSTM1* KO2 HepG2 cells, respectively, and the “HepG2 + plx-sg10-G7” single cell line was selected as the control group, named *SQSTM1* WT HepG2 cells.

### 3.3. SQSTM1/p62 Promotes the Migration and Invasion of HCC In Vitro

**Figure 3:**
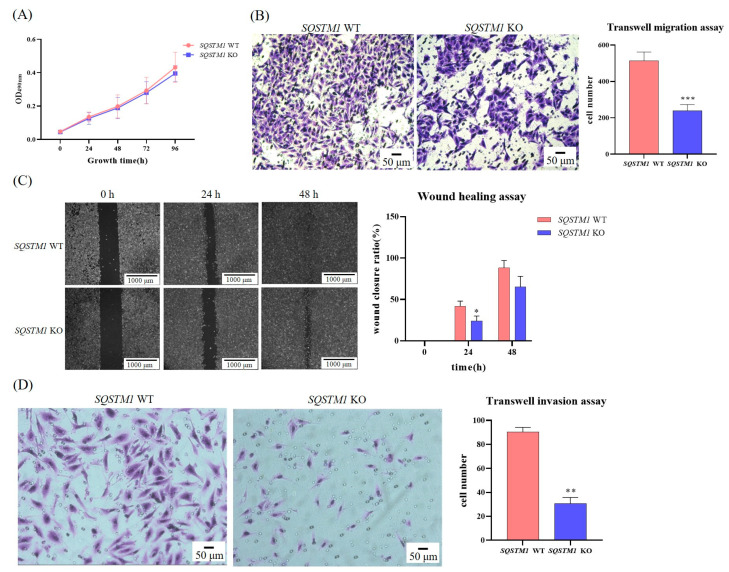
Effects of SQSTM1/p62 on migration and invasion of HCC in vitro. (**A**) Effects of SQSTM1/p62 on the proliferation of *SQSTM1* WT HepG2 cells and *SQSTM1* KO HepG2 cells were detected by drawing the growth curve. (**B**,**C**) Effects of SQSTM1/p62 on the migration ability of *SQSTM1* WT HepG2 cells and *SQSTM1* KO HepG2 cells were detected by Transwell migration assay (200×) and wound healing assay (50×). (**D**) Effects of SQSTM1/p62 on the invasiveness of *SQSTM1* WT HepG2 cells and *SQSTM1* KO HepG2 cells were detected by Transwell invasion assay (200×). All data are shown as mean ± SD (*n* = 3), * *p* < 0.05, ** *p* < 0.01, and *** *p* < 0.001.

**Figure A1:**
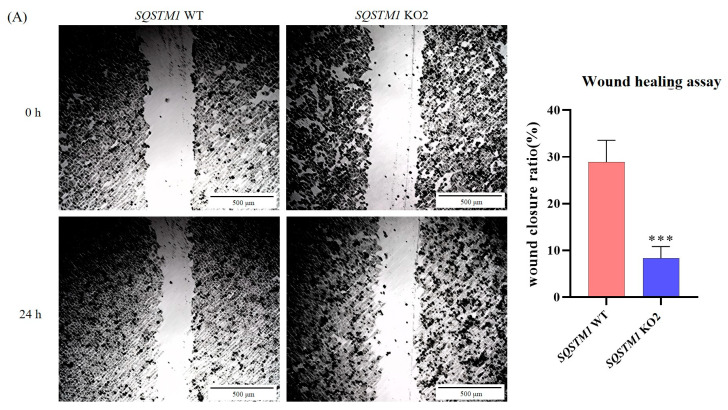

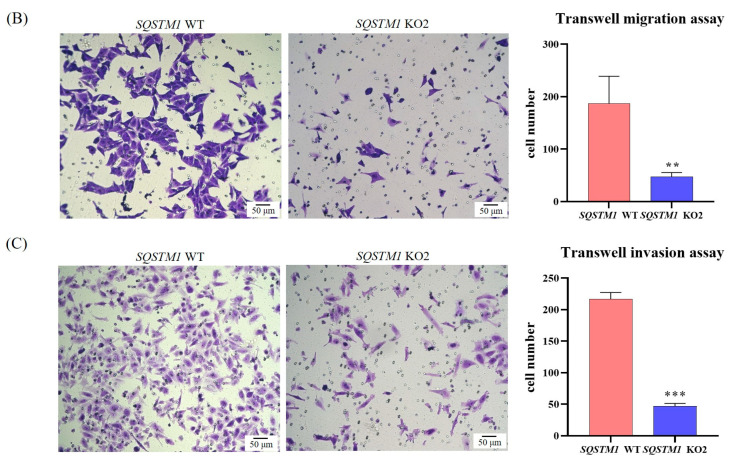
Effects of SQSTM1/p62 on the migration and invasion of HCC in vitro. (**A**) Effects of SQSTM1/p62 on the migration ability of *SQSTM1* WT HepG2 cells and *SQSTM1* KO2 HepG2 cells are detected by wound healing assay (100×). (**B**) Effects of SQSTM1/p62 on the migration ability of *SQSTM1* WT HepG2 cells and *SQSTM1* KO2 HepG2 cells are detected by Transwell migration assay (200×). (**C**) Effects of SQSTM1/p62 on the invasiveness of *SQSTM1* WT HepG2 cells and *SQSTM1* KO2 HepG2 cells were detected by Transwell invasion assay (200×). All data are shown as mean ± SD (*n* = 3), ** *p* < 0.01, and *** *p* < 0.001.

**Figure A2:**
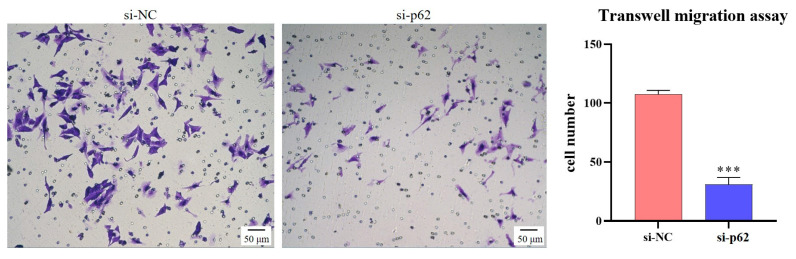
Effects of siRNA-p62 on the migration of HCC in vitro. Effects of siRNA-p62 on the migration of HCC are measured by Transwell migration assay (200×). All data are shown as mean ± SD (*n* = 3) and *** *p* < 0.001.

To confirm whether *SQSTM1/p62* knockout affected the survival of HepG2 cells, we used the MTT assay to determine the growth curves of *SQSTM1* WT HepG2 cells and *SQSTM1* KO HepG2 cells. The growth curves of *SQSTM1* WT HepG2 cells and *SQSTM1* KO HepG2 cells were not significantly different (Figure 3A). Therefore, it was concluded that the knockout of *SQSTM1/p62* had no significant impact on the survival of HepG2 cells. Next, we conducted wound healing, Transwell migration, and Transwell invasion assays on *SQSTM1* WT HepG2 cells and *SQSTM1* KO HepG2 cells to evaluate the influence of SQSTM1/p62 on the migration and invasion of HCC. The results showed that the scratch width of *SQSTM1* KO HepG2 cells was significantly greater than that of *SQSTM1* WT HepG2 cells at 24 h (Figure 3C), and the number of migrating cells (Figure 3B) and invasive cells (Figure 3D) was significantly lower than that of *SQSTM1* WT HepG2 cells at 24 h. In addition, considering that crispred out genes might show clonal variation with their function, we used another clonal cell (*SQSTM1* KO2 HepG2 cells) to perform wound healing, Transwell migration, and Transwell invasion assays. We found that *SQSTM1/p62* knockout showed similar inhibitory effects in *SQSTM1* KO2 HepG2 cells (Figure A1). Additionally, to eliminate the impact of the off-target or non-specific knockout or knockdown, we also transfected cells with siRNA-p62 to complete the Transwell migration assay and found that after the addition of siRNA-p62, the migration ability of cells became significantly weaker (Figure A2). These results suggest that *SQSTM1/p62* knockout significantly inhibited migration and invasion, indicating that SQSTM1/p62 might promote the migration and invasion of HCC and could be a poor prognostic factor.

### 3.4. SQSTM1/p62 Might Regulate HCC Migration and Invasion through the Keap1/Nrf2/MMP2 Signaling Pathway In Vitro

**Figure 4:**
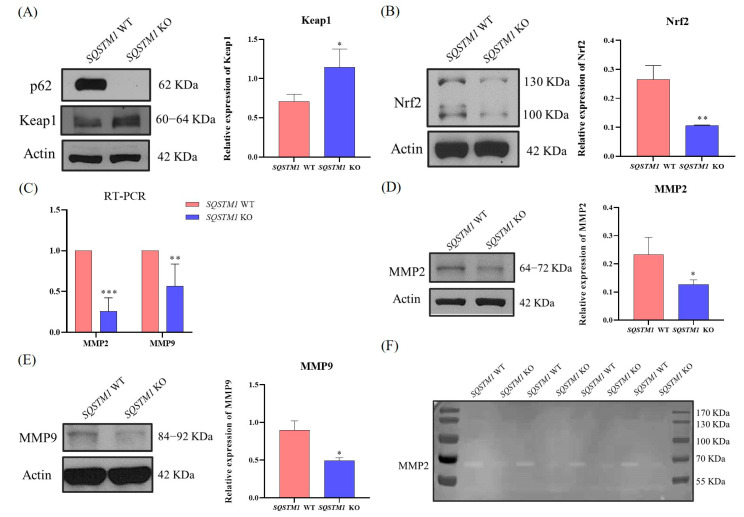
Molecular mechanism of SQSTM1/p62 regulating migration and invasion of human hepatoma cells HCC in vitro. (**A**,**B**) Effects of SQSTM1/p62 on the expression levels of Keap1 (**A**) and Nrf2 (**B**) in *SQSTM1* WT HepG2 cells and *SQSTM1* KO HepG2 cells were measured by WB. (**C**) Effects of SQSTM1/p62 on the mRNA levels of MMP2 and MMP9 in *SQSTM1* WT HepG2 cells and *SQSTM1* KO HepG2 cells were quantified by RT-PCR. (D-E) Effects of SQSTM1/p62 on the expression levels of MMP2 (**D**) and MMP9 (**E**) in *SQSTM1* WT HepG2 cells and *SQSTM1* KO HepG2 cells were measured by WB. (**F**) Effects of SQSTM1/p62 on the activity of MMP2 in *SQSTM1* WT HepG2 cells and *SQSTM1* KO HepG2 cells were detected by gelatin zymography assay. All data are represented as mean ± SD (*n* = 3), * *p* < 0.05, ** *p* < 0.01, and *** *p* < 0.001.

**Figure A3:**
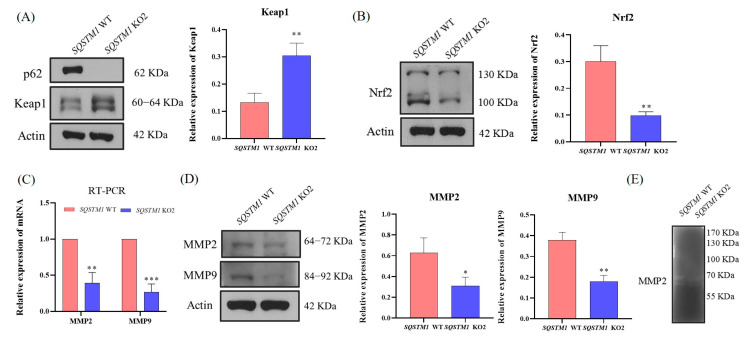
Molecular mechanism of SQSTM1/p62 regulating migration and invasion of HCC in vitro. (**A**,**B**) Effects of SQSTM1/p62 on the expression levels of Keap1 (**A**) and Nrf2 (**B**) in *SQSTM1* WT HepG2 cells and *SQSTM1* KO2 HepG2 cells were measured by WB. (**C**) Effects of SQSTM1/p62 on the mRNA levels of MMP2 and MMP9 in *SQSTM1* WT HepG2 cells and *SQSTM1* KO2 HepG2 cells were quantified by RT-PCR. (**D**) Effects of SQSTM1/p62 on the expression levels of MMP2 and MMP9 in *SQSTM1* WT HepG2 cells and *SQSTM1* KO2 HepG2 cells were measured by WB. (**E**) Effects of SQSTM1/p62 on the activity of MMP2 in *SQSTM1* WT HepG2 cells and *SQSTM1* KO2 HepG2 cells were detected by gelatin zymography assay. All data are represented as mean ± SD (*n* = 3), * *p* < 0.05, ** *p* < 0.01, and *** *p* < 0.001.

**Figure A4:**
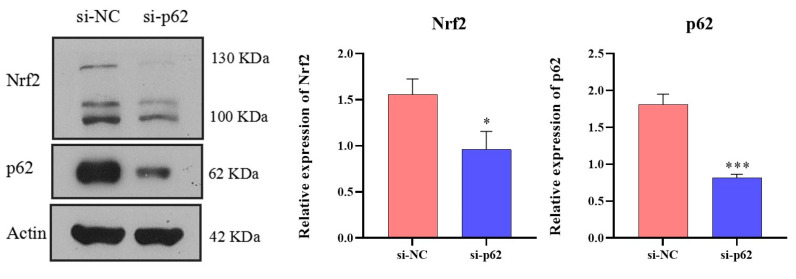
Molecular mechanism of siRNA-p62 regulating migration and invasion of HCC in vitro. The effect of siRNA-p62 on the expression level of Nrf2 was quantified by WB. All data are represented as mean ± SD (*n* = 3), * *p* < 0.05, and *** *p* < 0.001.

To further confirm the effect of SQSTM1/p62 on the migration and invasion of HCC cells, we used the *SQSTM1/p62* knockout cell lines (*SQSTM1* KO HepG2 cells and *SQSTM1* KO2 HepG2 cells) and found that after *SQSTM1/p62* knockdown, the expression level of Keap1 was significantly increased (Figure 4A and Figure A3A), while the expression level of Nrf2 was significantly decreased (Figure 4B and Figure A3B). Moreover, RT-PCR and gelatin zymography assays further showed that after *SQSTM1/p62* was knocked out, the mRNA expression level (Figure 4C and Figure A3C), the protein expression level (Figure 4D,E and Figure A3D), and activity (Figure 4F and Figure A3E) of MMP2 and MMP9 were significantly reduced. In addition, we also found similar results after siRNA-p62 (Figure A4), eliminating the effects of off-target or non-specific knockout or knockdown. Therefore, we preliminarily hypothesized that in HCC, loss of SQSTM1/p62 function led to the inactivation of Nrf2 by concealing the inhibitory effect of SQSTM1/p62 on Keap1, as Keap1 could specifically inhibit Nrf2 function. Inactivation of Nrf2 could inhibit the expression of matrix metalloproteinases MMP2 and MMP9 and attenuate ECM degradation. In other words, SQSTM1/p62 may promote the migration and invasion of HCC via the Keap1/Nrf2/MMP2 signaling pathway.

### 3.5. SQSTM1/p62 Regulates the Migration and Invasion of HCC through the Keap1/Nrf2/MMP2 Signaling Pathway In Vitro

**Figure 5:**
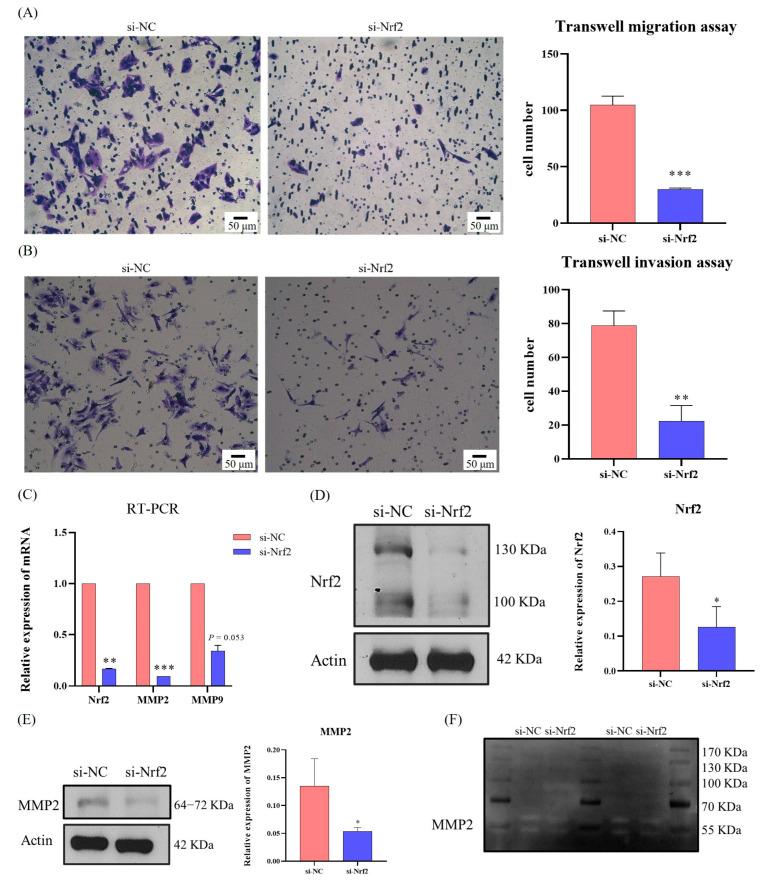
Verify whether SQSTM1/p62 regulated the migration and invasion of HCC through the Keap1/Nrf2/MMP2 signaling pathway in vitro. (**A**,**B**) Effects of siRNA-Nrf2 on migration and invasion of HCC were, respectively, measured by Transwell migration assay (200×) and Transwell invasion assay (200×). (**C**) Effects of siRNA-Nrf2 on mRNA levels of MMP2 and MMP9 were quantified by RT-PCR. (**D**,**E**) The effect of siRNA-Nrf2 on the expression level of MMP2 was quantified by WB. (**F**) The effect of siRNA-Nrf2 on the activity of MMP2 was detected by gelatin zymography assay. si-NC represents siRNA scramble control. All data are represented as mean ± SD (*n* = 3), * *p* < 0.05, ** *p* < 0.01, and *** *p* < 0.001.

To verify whether SQSTM1/p62 regulates the migration and invasion of HCC through the Keap1/Nrf2/MMP2 signaling pathway, we transfected cells with siRNA-Nrf2, a key transcription factor. As shown in Figure 5A–F, siRNA-Nrf2 transfection resulted in the significant inhibition of the migration (Figure 5A) and invasion ability (Figure 5B) of the cells. Furthermore, the mRNA expression level (Figure 5C), protein expression level (Figure 5D,E), and activity (Figure 5F) of MMP2 were also significantly reduced. Since SQSTM1/p62 can directly regulate Nrf2, the results of Nrf2 silencing further indicate that SQSTM1/p62 indeed regulates the migration and invasion of HCC through the Keap1/Nrf2/MMP2 signaling pathway.

### 3.6. SQSTM1/p62 Promotes the Migration and Invasion of HCC In Vivo

**Figure 6:**
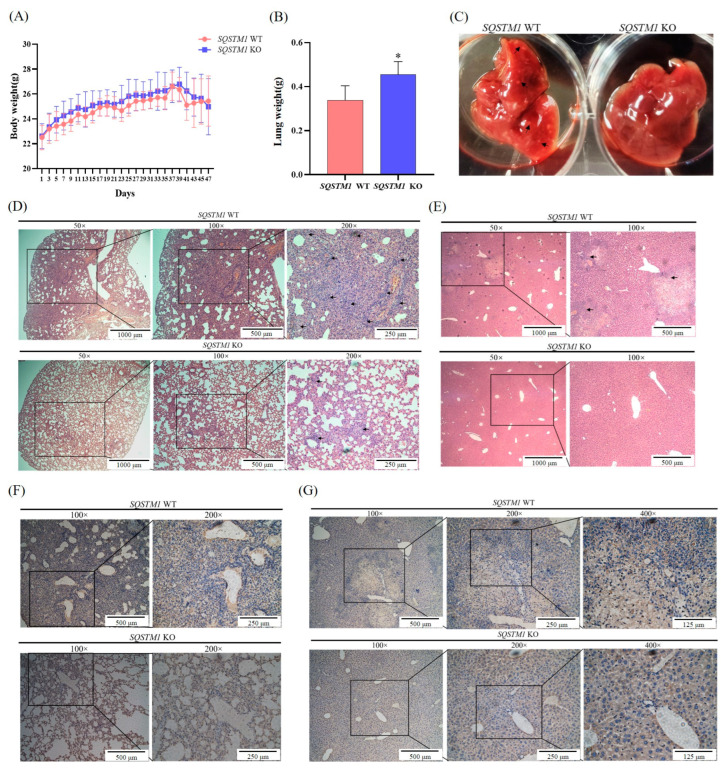
Effects of SQSTM1/p62 on migration and invasion of HCC in vivo. (**A**) Body weight, (**B**) lung weight, (**C**) distribution of metastatic lesions on the liver, (**D**,**E**) hematoxylin and eosin stained microsection of the lung (**D**) and liver (**E**) (black arrows indicate tumors), and (**F**,**G**) immunohistochemical analysis of MMP2 in the lung (**F**) and liver (**G**) of nude mice inoculated with *SQSTM1* WT HepG2 cells and *SQSTM1* KO HepG2 cells by tail vein injection for 7 weeks. All data are shown as mean ± SD (*n* = 5), * *p* < 0.05.

The in vitro cell experiments indicated that the knockout of *SQSTM1/p62* significantly inhibited the migration and invasion of HCC cells. Therefore, a lung metastasis model of liver cancer in nude mice was established by injectin*g SQSTM1* WT HepG2 cells or *SQSTM1* KO HepG2 cells into the tail vein to evaluate and verify the effects of SQSTM1/p62 on the migration and invasion of HCC cells in vivo. The results are presented in Figure 6A–C below. The body weight of nude mice in the *SQSTM1* WT group tended to decrease, the lung weight became lighter, and many metastatic foci were found in the liver after 7 weeks. The body and lung weights of nude mice in the *SQSTM1* KO group were relatively normal, and no metastatic foci were found in the liver. Furthermore, hematoxylin and eosin staining of the lung (Figure 6D) and liver (Figure 6E) showed that compared with the *SQSTM1* WT group, the lung tissue cells in the *SQSTM1* KO group were arranged more regularly, the number of tumor cells was decreased, and the degree of deterioration was lower, while no tumor was found in the liver. In addition, immunohistochemical analysis of MMP2 was performed using pathological sections. As shown in Figure 6F,G, staining in the *SQSTM1* KO group was lighter, indicating reduced expression of MMP2. These results indicated that SQSTM1/p62 promotes the migration and invasion of HCC cells in vivo. That is, *SQSTM1/p62* knockout reduced the expression of MMP2 and significantly slowed down the process of migration and invasion of HCC, which was consistent with the results of in vitro cell experiments.

### 3.7. DDP Inhibits Migration and Invasion of HCC Based on SQSTM1/p62 Target

**Figure 7:**
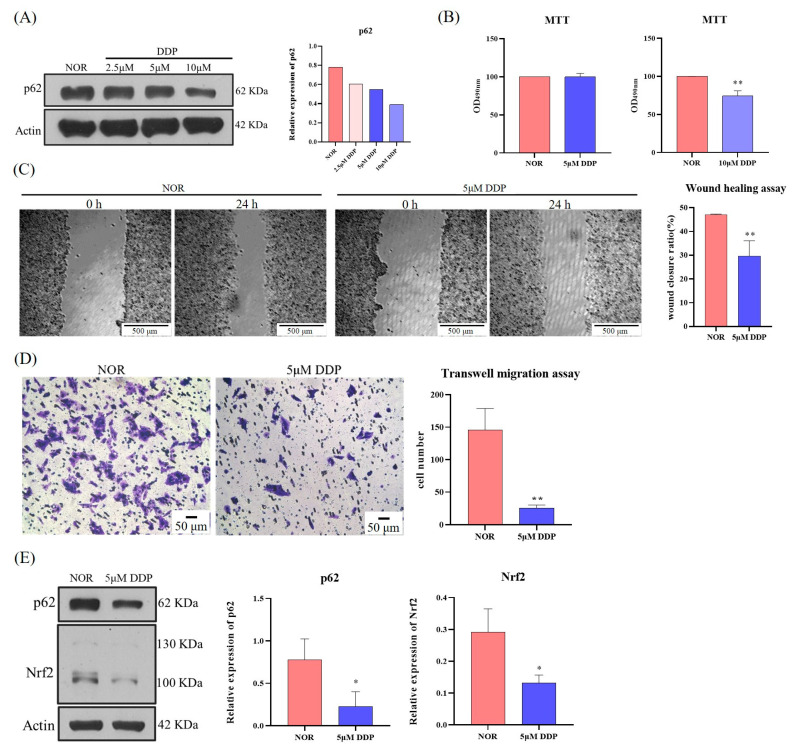
DDP inhibited migration and invasion of HCC based on the SQSTM1/p62 target. (**A**) Effects of DDP on the expression level of SQSTM1/p62 in *SQSTM1* WT HepG2 cells at final concentrations of 0–10 µM for 24 h were evaluated by WB. (**B**) Cytotoxicity of DPP (5 µM and 10 µM) on *SQSTM1* WT HepG2 cells for 24 h was evaluated by MTT assay. (**C**,**D**) Effects of 5 µM DDP on the migration ability of *SQSTM1* WT HepG2 cells were evaluated by wound healing assay (100×) (**C**) and Transwell migration assay (200×) (**D**). (**E**) Effects of 5 µM DDP on the expression levels of SQSTM1/p62 and Nrf2 in *SQSTM1* WT HepG2 cells were detected by WB. All data are represented as mean ± SD (*n* = 3), * *p* < 0.05, and ** *p* < 0.01.

This study demonstrated that SQSTM1/p62 is involved in the migration and invasion of HCC cells through the Keap1/Nrf2/MMP2 signaling pathway in vivo and in vitro. Whether certain drugs can regulate SQSTM1/p62 expression and exert an effect on the migration and invasion of HCC remains unclear. Therefore, we searched for drugs effectively targeting SQSTM1/p62 and found that the anti-tumor drug DDP significantly reduced the expression level of SQSTM1/p62 in *SQSTM1* WT HepG2 cells at 2.5–10 µM (Figure 7A). DDP at 10 µM showed obvious cytotoxicity; however, DDP at 5 µM had no significant effect on cell survival (Figure 7B). Therefore, 5 µM of DDP was selected for further experiments. As shown in Figure 7C,D, 5 µM DDP significantly reduced the rate of cell scratch healing (Figure 7C) and the number of migrated cells (Figure 7D) compared to the normal group. At the molecular level (Figure 7E), 5 µM DDP significantly reduced the expression of SQSTM1/p62 and Nrf2. These results indicate that DDP inhibits the migration and invasion of HCC, likely by lowering SQSTM1/p62 levels, providing mechanistic support for clinical treatment.

### 3.8. SQSTM1/p62 Promotes the Migration and Invasion of HCC More Vigorously in the Inflammatory Microenvironment

**Figure 8:**
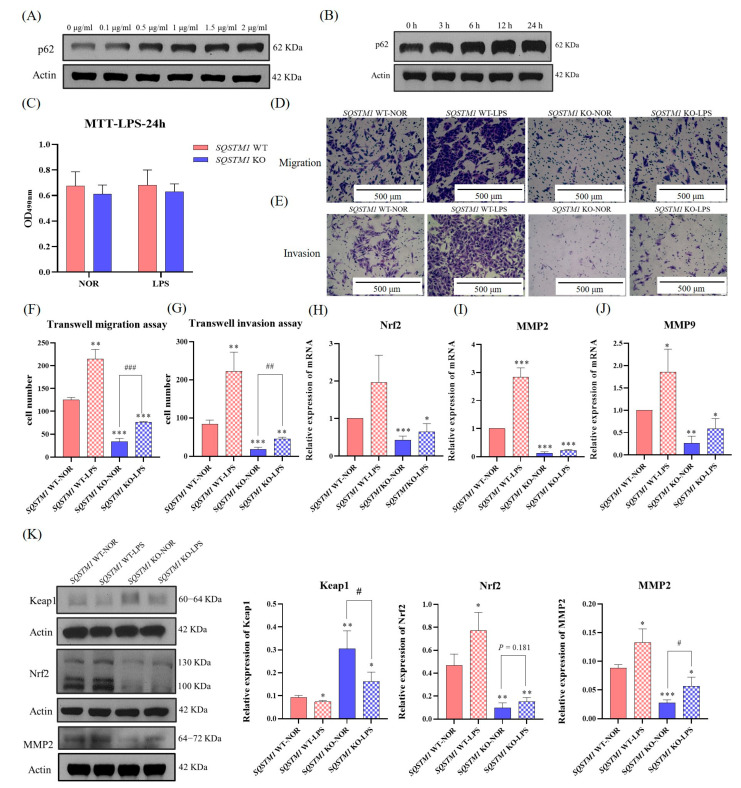
The effects and molecular mechanisms of SQSTM1/p62 on migration and invasion of HCC in the inflammatory microenvironment simulated by LPS. (**A**,**B**) Determination of the appropriate concentration and duration of LPS for simulating inflammatory microenvironment. Among them, effects of LPS on the expression level of SQSTM1/p62 in *SQSTM1* WT HepG2 cells at final concentrations of 0–2 µg/mL for 24 h were detected first, and then effects of LPS (1 µg/mL) on the expression level of SQSTM1/p62 in *SQSTM1* WT HepG2 cells after 0–24 h were detected. (**C**) Cytotoxicity of LPS (1 µg/mL) on *SQSTM1* WT HepG2 cells and *SQSTM1* KO HepG2 cells for 24 h were evaluated by MTT assay. (**D**–**G**) Effects of SQSTM1/p62 on migration and invasion of *SQSTM1* WT HepG2 cells and *SQSTM1* KO HepG2 cells in the inflammatory microenvironment were measured by Transwell migration assay (200×) (**D**,**F**) and Transwell invasion assay (200×) (**E**,**G**). (**H**–**J**) Effects of SQSTM1/p62 on the mRNA levels of Nrf2 (**H**), MMP2 (**I**), and MMP9 (**J**) in *SQSTM1* WT HepG2 cells and *SQSTM1* KO HepG2 cells in the inflammatory microenvironment were quantified by RT-PCR. (**K**) Effects of SQSTM1/p62 on the expression levels of Keap1, Nrf2, and MMP9 in *SQSTM1* WT HepG2 cells and *SQSTM1* KO HepG2 cells in the inflammatory microenvironment were detected by WB. All data are expressed as mean ± SD (*n* = 3), * *p* < 0.05, ** *p* < 0.01, and *** *p* < 0.001 versus *SQSTM1* WT NOR; # *p* < 0.05, ## *p* < 0.01, and ### *p* < 0.001 versus *SQSTM1* KO NOR.

**Figure A5:**
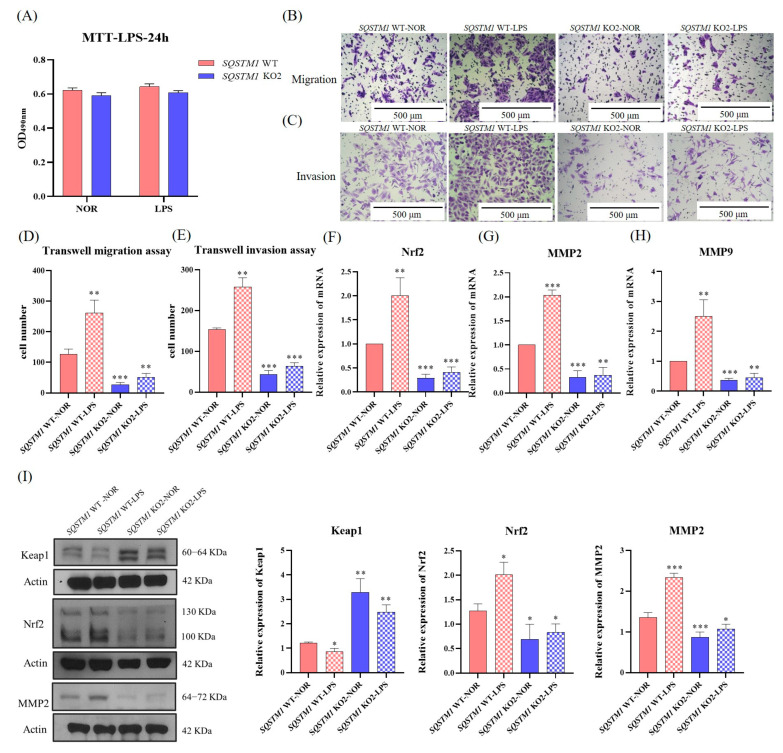
The effects and molecular mechanisms of SQSTM1/p62 on migration and invasion of HCC in the inflammatory microenvironment simulated by LPS. (**A**) Cytotoxicity of LPS (1 µg/mL) on *SQSTM1* WT HepG2 cells and *SQSTM1* KO2 HepG2 cells for 24 h were evaluated by MTT assay. (**B**–**E**) Effects of SQSTM1/p62 on migration and invasion of *SQSTM1* WT HepG2 cells and *SQSTM1* KO2 HepG2 cells in the inflammatory microenvironment were measured by Transwell migration assay (200×) (**B**,**D**) and Transwell invasion assay (200×) (**C**,**E**). (**F**–**H**) Effects of SQSTM1/p62 on the mRNA levels of Nrf2 (**F**), MMP2 (**G**), and MMP9 (**H**) in *SQSTM1* WT HepG2 cells and *SQSTM1* KO2 HepG2 cells in the inflammatory microenvironment were quantified by RT-PCR. (**I**) Effects of SQSTM1/p62 on the expression levels of Keap1, Nrf2, and MMP9 in *SQSTM1* WT HepG2 cells and *SQSTM1* KO2 HepG2 cells in the inflammatory microenvironment were detected by WB. All data are expressed as mean ± SD (*n* = 3), * *p* < 0.05, ** *p* < 0.01, and *** *p* < 0.001 versus *SQSTM1* WT NOR.

**Figure A6:**
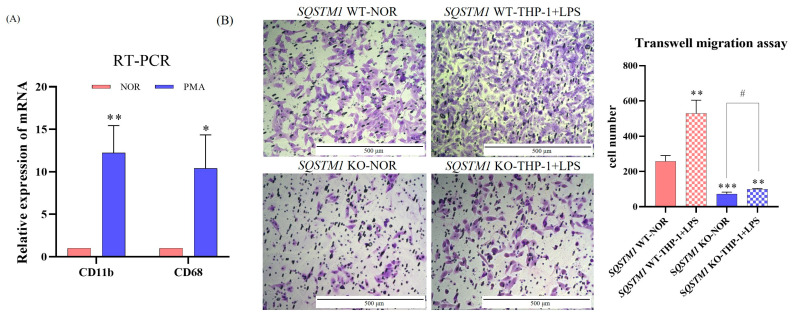
The effects of SQSTM1/p62 on the migration of HCC in the LPS-induced THP-1 macrophage inflammatory model. (**A**) The mRNA expression levels of CD11b and CD68 in THP-1 monocytes induced by 100 ng/mL PMA for 48 h are quantified by RT-PCR. (**B**) The effects of SQSTM1/p62 on the migration of *SQSTM1* WT HepG2 cells and *SQSTM1* KO HepG2 cells in the THP-1 macrophage inflammatory medium are measured by Transwell migration assay (200×). All data are expressed as mean ± SD (*n* = 3), * *p* < 0.05, ** *p* < 0.01, and *** *p* < 0.001 versus *SQSTM1* WT NOR; # *p* < 0.05 versus *SQSTM1* KO NOR.

The inflammatory microenvironment has a certain impact on tumor progression, and it can also regulate the expression of SQSTM1/p62 via the NF-κB signaling pathway [27,28,29]. Therefore, to simulate the inflammatory microenvironment, *SQSTM1* WT HepG2 cells were treated with 1 µg/mL LPS (Figure 8A–C), and their migration and invasion abilities were investigated. As shown in Figure 8A–K, treatment of *SQSTM1* WT HepG2 cells with 1 µg/mL LPS significantly increased the expression of SQSTM1/p62 (Figure 8A,B) and the number of migrating (Figure 8D,F) and invading cells (Figure 8E,G). At the mRNA and protein levels, the expression of Keap1 (Figure 8K) was significantly reduced, and the expression of Nrf2 (Figure 8H,K), MMP2 (Figure 8I,K), and MMP9 (Figure 8J) was significantly increased. However, treatment of *SQSTM1* KO HepG2 cells with LPS resulted in significantly decreased effects compared to the wild-type controls. We found similar results in another clonal cell (*SQSTM1* KO2 HepG2 cells) (Figure A5). In addition, considering the complexity of the tumor microenvironment (TME), we also studied the effects of SQSTM1/p62 on the migration of HCC in the LPS-induced THP-1 macrophage inflammatory model. As shown in Figure A6, we first induced THP-1 monocytes with 100 ng/mL phorbol ester (phorbol-12-myristate-13-acetate, PMA). After 48 h, we found that THP-1 monocytes had adhered to the wall, and the mRNA levels of CD11 and CD68 (important markers of macrophages) in THP-1 were significantly increased, indicating that THP-1 monocytes had successfully differentiated into macrophages. Therefore, we used 1 µg/mL LPS to stimulate THP-1 macrophages to construct an inflammatory model and then took its supernatant to act on *SQSTM1* WT HepG2 cells and *SQSTM1* KO HepG2 cells. Finally, through the Transwell migration experiment, we found that compared to “*SQSTM1* WT-NOR”, the number of migrating cells in *SQSTM1* WT HepG2 cells treated with the THP-1 macrophage inflammatory medium was significantly increased. Taken together, these results further showed that SQSTM1/p62 has a great impact on the migration and invasion of HCC, and pathological or pharmacological regulation of SQSTM1/p62 could be a key strategy in the treatment of HCC in the future.

## 4. Discussion

HCC is the sixth most common malignant tumor worldwide, with high mortality and invasiveness [1,43]. Although various treatment methods for HCC have been developed and improved [44], the survival rate and recurrence of HCC have not been properly resolved owing to the inconspicuous clinical symptoms at the early stage of HCC. Extensive research has found that migration and invasion are signs of HCC entering the late stage and seriously affect the success rate of HCC treatment [6,7]. Therefore, inhibiting the migration and invasion of HCC cells is the main trend of current research in the field of HCC treatment.

This study first found that SQSTM1/p62 was highly expressed in tumor tissues with strong invasion and migration ability, especially in HCC tissues, through the analysis of large data of clinical samples. It was hypothesized that SQSTM1/p62 plays an important role in these processes. Recently, many studies have confirmed that SQSTM1/p62 regulates the occurrence and development of various tumors [13,14,15,16], but most studies have focused on autophagy. However, in HCC, whether SQSTM1/p62 regulates tumor migration and invasion and the mechanisms involved remain unclear. Therefore, in this study, we constructed *SQSTM1* WT HepG2 cells and *SQSTM1* KO HepG2 cells using the CRISPR/Cas9 system, studied the effects of SQSTM1/p62 on migration and invasion of HCC cells in vivo and in vitro, and determined whether SQSTM1/p62 can serve as an important target for cancer treatment.

In this study, we first showed that *SQSTM1/p62* knockout could significantly inhibit the migration and invasion of HCC cells using wound healing and Transwell assays. SQSTM1/p62 can promote Keap1 degradation and cancel the inhibition activity on Nrf2. So, SQSTM1/p62 can promote Nrf2 activity. Recent studies have shown that Nrf2 overexpression can mediate the survival and development of cancer cells through multiple signaling pathways [45,46]. Nrf2 is highly expressed in HCC and plays an inhibitory role in the initial stage of hepatocarcinogenesis. However, with the gradual progression of liver cancer, Nrf2 will promote the expression of MMP9 [19] and is a key factor in promoting the proliferation, metastasis, invasion, and other malignant biological behaviors of HCC [47]. We suspected that SQSTM1/p62 might act in the progression of HCC through the Keap1/Nrf2/MMP2 signaling pathway, but this requires further validation.

Further experiments showed that in HCC, *SQSTM1/p62* knockout resulted in a significant increase in the expression of Keap1 and a significant reduction in the expression of Nrf2. Furthermore, the mRNA and protein levels of MMP2 and MMP9 were significantly decreased. MMP2 is a Zn^2+^-dependent proteolytic enzyme that hydrolyzes type IV collagen, activates MMP9 to degrade ECM, helps tumor cells break through the basement membrane, and plays a very important role in tumor migration and invasion [48,49,50,51]. Relevant experiments indicated that the enzymatic activity of MMP2 was significantly reduced, which further clarified the molecular mechanism of inhibiting the migration and invasion of HCC after *SQSTM1/p62* knockout. Furthermore, we verified that after Nrf2 silencing, the expression and activity of MMP2 declined significantly, indicating that Nrf2 affects MMP2 activity. Through an in vivo study, we also found that *SQSTM1/p62* knockout affected HCC migration, and the mechanisms might be associated with the Nrf2/MMP pathway. Therefore, this study demonstrated that SQSTM1/p62 could promote the migration and invasion of HCC cells through the Keap1/Nrf2/MMP2 signaling pathway.

SQSTM1/p62 appears to mediate the migration and invasion of tumors and may serve as a promising target for drug treatment. However, drugs that regulate SQSTM1/p62 remain unclear. Therefore, we searched for relevant drugs targeting SQSTM1/p62. Interestingly, this study revealed that DDP, an antitumor drug, significantly inhibited the expression of SQSTM1/p62 and slowed the migration and invasion of HCC cells. According to the domestic literature, there have been many reports on the impact of cisplatin on tumor occurrence and development, but they mainly focus on drug resistance, cytotoxicity, inhibition of proliferation, and apoptosis [52,53,54,55]. However, there are no reports on the impact of DDP on the migration and invasion of HCC cells through SQSTM1/p62. Therefore, another novelty of this study is that it suggests a new treatment strategy to inhibit the migration and invasion of HCC.

In addition, emerging evidence indicates that the inflammatory microenvironment plays an important role in tumor progression [25]. Several studies have reported that SQSTM1/p62 is significantly upregulated in the inflammatory microenvironment [26,27,28,29]. Therefore, this study further explored the impact and mechanism of SQSTM1/p62 on the migration and invasion of HCC cells in an inflammatory microenvironment. It was found that the inflammatory microenvironment simulated by LPS treatment upregulated SQSTM1/p62 and significantly affected the migration and invasion of HCC. This effect of inflammation on the migration and invasion of HCC seems to depend on SQSTM1/p62, but further validation is required. Taken together, the pharmacological or pathological changes in SQSTM1/p62 levels can affect the migration and invasion of HCC cells. 

**Figure 9:**
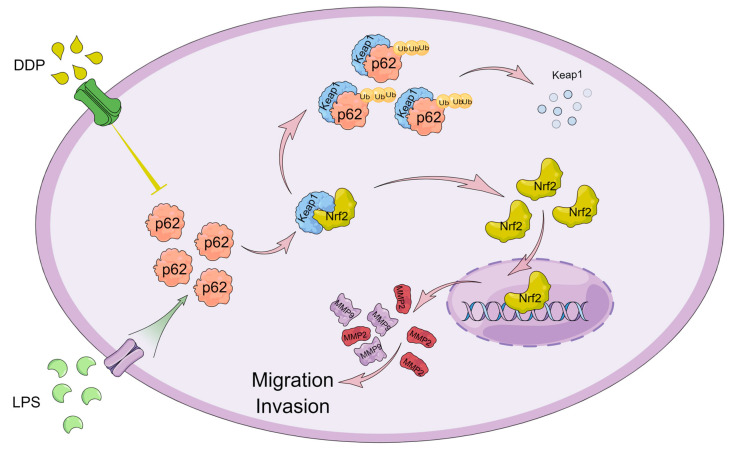
Effects and mechanisms of SQSTM1/p62 on migration and invasion of HCC. SQSTM1/p62 binds to Keap1 and leads to the ubiquitin-mediated degradation of Keap1, and then weakens the specific inhibitory effect of Keap1 on Nrf2. So, Nrf2 is activated and enters into the nucleus and then promotes the expression of MMP2 and MMP9, thereby promoting the migration and invasion of HCC. That is, SQSTM1/p62 promotes the migration and invasion of HCC through the Keap1/Nrf2/MMP2 signaling pathway. Knockout of *SQSTM1/p62* in HCC using the CRISPR/Cas9 system shows an attenuated migration and invasion in this manuscript. In addition, the pharmacological (DDP) or pathological (LPS) regulation in SQSTM1/p62 levels can affect the migration and invasion of HCC cells. So, SQSTM1/p62 plays a key role in the migration and invasion of HCC and may be developed into an effective intervention target for cancer cell migration and invasion.

In conclusion, in this study, we first found that SQSTM1/p62 plays a key role in the migration and invasion of HCC in vitro and in vivo using the CRISPR/Cas9 system (Figure 9). *SQSTM1/p62* knockout may inhibit the migration and invasion of HCC through the Keap1/Nrf2/MMP2 signaling pathway. The pharmacological or pathological regulation of SQSTM1/p62 can affect the migration and invasion of HCC cells (Figure 9). This study indicated that SQSTM1/p62 is a promising target for the treatment of HCC migration and invasion. However, considering that only one cell line (HepG2) is used in the functional experiments in this study, which has certain limitations, further validation studies should be conducted in the future.

## Data Availability

All core data supporting the results of this study are available within the article. Data can be provided upon a reasonable request.

## Appendix A

**Table A1:** Primers required for the construction of recombinant plasmid.

Primers	Primer Sequence (5′-3′)
R1-h-p62-2	TGTCCTACGTGAAGGATGACGGTGTTTCGTCCTTTCC
F2-h-p62-2	GTCATCCTTCACGTAGGACAGTTTTAGAGCTAGAAATAGCAA
R1-h-p62-3	ACGTGAAGGATGACATCTTCGGTGTTTCGTCCTTTCC
F2-h-p62-3	GAAGATGTCATCCTTCACGTGTTTTAGAGCTAGAAATAGCAA
R1-h-p62-8	ACCGTGTGCTCAGGAGGCGCGGTGTTTCGTCCTTTCC
F2-h-p62-8	GCGCCTCCTGAGCACACGGTGTTTTAGAGCTAGAAATAGCAA
R1-h-p62-10	TTGCAGCCATCGCAGATCACGGTGTTTCGTCCTTTCC
F2-h-p62-10	GTGATCTGCGATGGCTGCAAGTTTTAGAGCTAGAAATAGCAA
F1	AAACTCGAGTGTACAAAAAAGCAGGCTTTAAAG
R2	AAAGCTAGCTAATGCCAACTTTGTACAAGAAAGCTG

**Table A2:** Preparation method of 5× loading buffer.

Reagent	Concentration	Manufacturer
Tris	60 mM	Sangon Biotech, China
Sodium dodecyl sulfate	2%	Sangon Biotech, China
Glycerine	10%	Sangon Biotech, China
Bromophenol blue	0.1%	Zhanyun Chemical Co., Ltd., Shanghai, China

**Table A3:** Preparation method of eluant (pH 7.6).

Reagent	Concentration	Manufacturer
Triton X-100	2.5%	Sangon Biotech, China
Tris	50 mM	Sangon Biotech, China
Calcium chloride	5 mM	Damao Chemical Reagent Co., Ltd., Tianjin, China
ZnCl_2_	0.001 mM	JinShi Chemistry Co., Ltd., Zhangye, China

**Table A4:** Preparation method of rinsing solution (pH 7.6).

Reagent	Concentration	Manufacturer
Tris	50 mM	Sangon Biotech, China
Calcium chloride	5 mM	Damao Chemical Reagent Co., Ltd., China
ZnCl_2_	0.001 mM	JinShi Chemistry Co., Ltd., China

**Table A5:** Preparation method of incubated buffer (pH 6.8).

Reagent	Concentration	Manufacturer
Tris	50 mM	Sangon Biotech, China
NaCl	200 mM	Sangon Biotech, China
Calcium chloride	5 mM	Damao Chemical Reagent Co., Ltd., China
Brij-35	0.02%	Sangon Biotech, China
ZnCl_2_	0.001 mM	JinShi Chemistry Co., Ltd., China

**Table A6:** Relationship between expression of SQSTM1/p62 and clinical factors in HCC.

Clinical Factors	Group	N	p62(−)	p62(+)	*p* Value
Tris	Female	10	6	4	>0.05
	Male	83	36	47	
Age	<50	30	15	15	>0.05
	≥50	63	27	36	
Tumor size	<5 cm	38	22	16	<0.05
	≥5 cm	55	20	35	
Histological grade	I–II	59	35	24	<0.01
	III–IV	34	7	27	
Venous invasion	absent	74	41	33	<0.01
	Present	19	1	18	
Metastasis	Negative	80	41	39	<0.01
	Positive	13	1	12	
TNM	I–II	33	20	13	<0.05
	III–IV	60	22	38	

## References

[1] Jacques F., Isabelle S., Rajesh D., Sultan E., Colin M., Marise R., Maxwell P.D., David F., Freddie B. (2015). Cancer incidence and mortality worldwide: Sources, methods and major patterns in GLOBOCAN 2012. Int. J. Cancer.

[2] Siegel R.L., Miller K.D., Jemal A. (2018). Cancer statistics, 2018. CA Cancer J. Clin..

[3] Zhong J., Sun P.B., Xu N.H., Liao M.J., Xu C.K., Ding Y.P., Cai J., Zhang Y.O., Xie W.D. (2020). Canagliflozin inhibits p-gp function and early autophagy and improves the sensitivity to the antitumor effect of doxorubicin. Biochem. Pharmacol..

[4] Yang J.D., Roberts L.R. (2010). Hepatocellular carcinoma: A global view. Nat. Rev. Gastroenterol. Hepatol..

[5] Forner A., Reig M., Bruix J. (2018). Hepatocellular carcinoma. Lancet.

[6] Zhang Y.S., Li D.D., Feng F., An L., Hui F.H., Dang D.S., Zhao Q.C. (2017). Progressive and prognosis value of notch receptors and ligands in hepatocellular carcinoma: A systematic review and meta-analysis. Sci. Rep..

[7] Valastyan S., Weinberg R.A. (2011). Tumor metastasis: Molecular insights and evolving paradigms. Cell.

[8] Colecchia A., Schiumerini R., Cucchetti A., Cescon M., Taddia M., Marasco G., Festi D. (2014). Prognostic factors for hepatocellular carcinoma recurrence. World J. Gastroenterol..

[9] Svenning S., Lamark T., Krause K., Johansen T. (2011). Plant NBR1 is a selective autophagy substrate and a functional hybrid of the mammalian autophagic adapters NBR1 and p62/SQSTM1. Autophagy.

[10] Shin J. (1998). P62 and the sequestosome, a novel mechanism for protein metabolism. Arch. Pharm. Res..

[11] Ning S.B., Wang L. (2019). The multifunctional protein p62 and its mechanistic roles in cancers. Curr. Cancer Drug. Targets.

[12] Moscat J., Karin M., Diaz-Meco M.T. (2016). p62 in cancer: Signaling adaptor beyond autophagy. Cell.

[13] Iwadate R., Inoue J., Tsuda H., Takano M., Furuya K., Hirasawa A., Aoki D., Inazawa J. (2015). High expression of p62 protein is associated with poor prognosis and aggressive phenotypes in endometrial cancer. Am. J. Pathol..

[14] Mao Y., Deng S.J., Su Y.J., Diao C., Peng Y., Ma J.F., Cheng R.C. (2021). The role of P62 in the development of human thyroid cancer and its possible mechanism. Cancer Genet..

[15] Wei Y.Z., Liu D.Y., Jin X.X., Gao P., Wang Q.Y., Zhang J.W., Zhang N. (2016). PA-MSHA inhibits the growth of doxorubicin-resistant MCF-7/ADR human breast cancer cells by downregulating Nrf2/p62. Cancer Med..

[16] Rolland P., Madjd Z., Durrant L., Ellis I.O., Layfield R., Spendlove I. (2007). The ubiquitin-binding protein p62 is expressed in breast cancers showing features of aggressive disease. Endocr. Relat. Cancer.

[17] Sun X.F., Ou Z.H., Chen R.C., Niu X.H., Chen D., Kang R., Tang D.L. (2016). Activation of the p62-Keap1-NRF2 pathway protects against ferroptosis in hepatocellular carcinoma cells. Hepatology.

[18] Lau A., Wang X.J., Zhao F., Villeneuve N.F., Wu T.D., Jiang T., Sun Z., White E., Zhang D.D. (2010). A noncanonical mechanism of Nrf2 activation by autophagy deficiency: Direct interaction between Keap1 and p62. Mol. Cell. Biol..

[19] Ngo H.K.C., Kim D.H., Suh J.Y., Park S.A., Kim S.J., Saeidi S., Na H.K., Surh Y.J. (2018). Differential roles for the redox sensitive transcription factor Nrf2 in carcinogenesis. Free Radic. Biol. Med..

[20] Lamoreaux W.J., Fitzgerald M.E.C., Reiner A., Hasty K.A., Charles S.T. (1998). Vascular endothelial growth factor increases release of gelatinase a and decreases release of tissue inhibitor of metalloproteinases by microvascular endothelial cells in vitro. Microvasc. Res..

[21] Hojilla C.V., Wood G.A., Khokha R. (2008). Inflammation and breast cancer: Metalloproteinases as common effectors of inflammation and extracellular matrix breakdown in breast cancer. Breast Cancer Res. BCR.

[22] Liubomirski Y., Lerrer S., Meshel T., Rubinstein-Achiasaf L., Morein D., Wiemann S., Körner C., Ben-Baruch A. (2019). Tumor-stroma-inflammation networks promote pro-metastatic chemokines and aggressiveness characteristics in triple-negative breast cancer. Front. Immunol..

[23] Mantovani A., Allavena P., Sica A., Balkwill F. (2008). Cancer-related inflammation. Nature.

[24] Sumimoto H., Imabayashi F., Yutaka Kawakami T.I. (2006). The BRAF-MAPK signaling pathway is essential for cancer-immune evasion in human melanoma cells. J. Exp. Med..

[25] Feller L., Altini M., Lemmer J. (2013). Inflammation in the context of oral cancer. Oral. Oncol..

[26] Yang H.H., Wang L.D., Yang M.S., Hu J.Q., Zhang E., Peng L.P. (2022). Oridonin attenuates LPS-induced early pulmonary fibrosis by regulating impaired autophagy, oxidative stress, inflammation and EMT. Eur. J. Pharmacol..

[27] Emanuele S., Lauricella M., D’Anneo A., Carlisi D., Blasio A.D., Liberto D.D., Giuliano M. (2020). p62: Friend or foe? evidences for oncoJanus and neuroJanus roles. Int. J. Mol. Sci..

[28] Yang S.W., Qiang L., Sample A., Shah P., He Y.Y. (2017). NF-κB signaling activation induced by chloroquine requires autophagosome, p62 protein, and c-Jun N-terminal Kinase (JNK) signaling and promotes tumor cell resistance. J. Biol. Chem..

[29] Zhong Z.Y., Umemura A., Sanchez-Lopez E., Liang S., Shalapour S., Wong J., He F., Boassa D., Perkins G., Raza Ali S. (2016). NF-kappa B restricts inflammasome activation via elimination of damaged mitochondria. Cell.

[30] Mirzaei S., Mohammadi A.T., Gholami M.H., Hashemi F., Zarrabi A., Zabolian A., Hushmandi K., Makvandi P., Samec M., Liskova A. (2021). Nrf2 signaling pathway in cisplatin chemotherapy: Potential involvement in organ protection and chemoresistance. Pharmacol. Res..

[31] Han X.J., Yang Z.J., Jiang L.P., Wei Y.F., Liao M.F., Qian Y.S., Li Y., Huang X., Wang J.B., Xin H.B. (2015). Mitochondrial dynamics regulates hypoxia-induced migration and antineoplastic activity of cisplatin in breast cancer cells. Int. J. Oncol..

[32] Wu H., Du C., Yang F., Zheng X., Qiu D., Zhang Q., Chen W., Xu Y. (2020). Generation of hepatocyte-like cells from human urinary epithelial cells and the role of autophagy during direct reprogramming. Biochem. Biophys. Res. Commun..

[33] Pai Bellare G., Saha B., Patro B.S. (2021). Targeting autophagy reverses de novo resistance in homologous recombination repair proficient breast cancers to PARP inhibition. Br. J. Cancer.

[34] Li T.W., Fu J.X., Zeng Z.X., Cohen D., Li J., Chen Q.M., Li B., Liu X.S. (2020). TIMER2.0 for analysis of tumor-infiltrating immune cells. Nucleic Acids Res..

[35] Tang Z.F., Li C.W., Kang B.X., Gao G., Li C., Zhang Z.M. (2017). GEPIA: A web server for cancer and normal gene expression profiling and interactive analyses. Nucleic Acids Res..

[36] Wang T., Wei J.J., Sabatini D.M., Lander E.S. (2014). Genetic screens in human cells using the CRISPR-Cas9 system. Science.

[37] Jiang X., Xu C., Lei F., Liao M., Wang W., Xu N., Zhang Y., Xie W. (2017). MiR-30a targets IL-1α and regulates islet functions as an inflammation buffer and response factor. Sci. Rep..

[38] Sun P.B., Wang Y.Y., Ding Y.P., Luo J.Y., Zhong J., Xu N.H., Zhang Y.O., Xie W.D. (2021). Canagliflozin attenuates lipotoxicity in cardiomyocytes and protects diabetic mouse hearts by inhibiting the mTOR/HIF-1α pathway. iScience.

[39] Ha K.T., Kim J.K., Kang S.K., Kim D.W., Lee Y.C., Kim H.M., Kim C.H. (2004). Inhibitory effect of Sihoga-Yonggol-Moryo-Tang on matrix metalloproteinase-2 and -9 activities and invasiveness potential of hepatocellular carcinoma. Pharmacol. Res..

[40] Xie W.D., Nie Y., Du L.J., Zhang Y.O., Cai G.P. (2007). Preventive effects of fenofibrate on insulin resistance, hyperglycaemia, visceral fat accumulation in NIH mice induced by small-dose streptozotocin and lard. Pharmacol. Res..

[41] Wu B. (2018). Expression and Clinical Significance of ADAM10 and p62 in Hepatocellular Carcinoma. Master’s Thesis.

[42] Brezgin S., Kostyusheva A., Kostyushev D., Chulanov V. (2019). Dead cas systems: Types, principles, and applications. Int. J. Mol. Sci..

[43] Cidon E.U. (2017). Systemic treatment of hepatocellular carcinoma: Past, present and future. World J. Hepatol..

[44] Komatsu M., Waguri S., Koike M., Sou Y.S., Ueno T., Hara T., Mizushima N., Iwata J.I., Ezaki J., Murata S. (2007). Homeostatic levels of p62 control cytoplasmic inclusion body formation in autophagy-deficient mice. Cell.

[45] Thijssen V.L., Paulis Y.W., Sliwinska P.N., Deumelandt K.L., Hosaka K., Soetekouw P.M., Cimpean A.M., Raica M., Pauwels P., Oord J.J.V.D. (2018). Targeting PDGF-mediated recruitment of pericytes blocks vascular mimicry and tumor growth. J. Pathol..

[46] DeNicola G.M., Karreth F.A., Humpton T.J., Gopinathan A., Wei C., Frese K., Mangal D., Yu K.H., Yeo C.J., Calhoun E.S. (2011). Oncogene-induced Nrf2 transcription promotes ROS detoxification and tumorigenesis. Nature.

[47] Kuźniak V.K., Paluszczak J., Dubowska W.B. (2017). The Nrf2-ARE signaling pathway: An update on its regulation and possible role in cancer prevention and treatment. Pharmacol. Rep..

[48] Kwiecien I., Stelmaszczyk-Emmel A., Polubiec-Kownacka M., Dziedzic D., Domagala-Kulawik J. (2017). Elevated regulatory T cells, surface and intracellular CTLA-4 expression and interleukin-17 in the lung cancer microenvironment in humans. Cancer Immunol. Immunother..

[49] Chang Y.H., Yu C.W., Lai L.C., Tsao C.H., Ho K.T., Yang S.C., Lee H., Cheng Y.W., Wu T.C., Shiau M.Y. (2010). Up-regulation of interleukin-17 expression by human papillomavirus type 16 E6 in nonsmall cell lung cancer. Cancer.

[50] Wang B., Liu T., Wu J.C., Luo S.Z., Chen R., Lu L.G., Xu M. (2018). STAT3 aggravates TGF-β1-induced hepatic epithelial-to-mesenchymal transition and migration. Biomed. Pharmacother..

[51] Duff D., Long A. (2017). Roles for RACK1 in cancer cell migration and invasion. Cell. Signal..

[52] Brozovic A., Ambriović-Ristov A., Osmak M. (2010). The relationship between cisplatin-induced reactive oxygen species, glutathione, and BCL-2 and resistance to cisplatin. Crit. Rev. Toxicol..

[53] Desoize B. (2002). Cancer and metals and metal compounds: Part I—carcinogenesis. Crit. Rev. Oncol. Hematol..

[54] Clodfelter J.E., Gentry M.B., Drotschmann K. (2005). MSH2 missense mutations alter cisplatin cytotoxicity and promote cisplatin-induced genome instability. Nucleic Acids Res..

[55] Sun X.P., Dong X., Lin L., Jiang X., Wei Z., Zhai B., Sun B., Zhang Q., Wan X., Jiang H. (2014). Up-regulation of survivin by AKT and hypoxia-inducible factor 1a contributes to cisplatin resistance in gastric cancer. FEBS J..

